# Emerging Roles of Lysyl Oxidases in the Cardiovascular System: New Concepts and Therapeutic Challenges

**DOI:** 10.3390/biom9100610

**Published:** 2019-10-14

**Authors:** José Martínez-González, Saray Varona, Laia Cañes, María Galán, Ana M Briones, Victoria Cachofeiro, Cristina Rodríguez

**Affiliations:** 1 Instituto de Investigaciones Biomédicas de Barcelona (IIBB-CSIC), 08036 Barcelona, Spain; jose.martinez@iibb.csic.es (J.M.-G.);; 2 CIBER de Enfermedades Cardiovasculares, Instituto de Salud Carlos III, 28029 Madrid, Spain; 3 Instituto de Investigación Biomédica Sant Pau (IIB-Sant Pau), 08041 Barcelona, Spain; 4 Institut de Recerca Hospital de la Santa Creu i Sant Pau-Programa ICCC, 08025 Barcelona, Spain; 5 Departmento de Farmacología, Facultad de Medicina, Universidad Autónoma de Madrid, Instituto de Investigación Hospital La Paz, 28029 Madrid, Spain; 6 Departamento de Fisiología, Facultad de Medicina, Universidad Complutense de Madrid-Instituto de Investigación Sanitaria Gregorio Marañón (IiSGM), 28040 Madrid, Spain

**Keywords:** lysyl oxidases, cardiovascular diseases, atherosclerosis, aortic aneurysm, vascular calcification, vascular stiffness, myocardial fibrosis

## Abstract

Lysyl oxidases (LOX and LOX-likes (LOXLs) isoenzymes) belong to a family of copper-dependent enzymes classically involved in the covalent cross-linking of collagen and elastin, a pivotal process that ensures extracellular matrix (ECM) stability and provides the tensile and elastic characteristics of connective tissues. Besides this structural role, in the last years, novel biological properties have been attributed to these enzymes, which can critically influence cardiovascular function. LOX and LOXLs control cell proliferation, migration, adhesion, differentiation, oxidative stress, and transcriptional regulation and, thereby, their dysregulation has been linked to a myriad of cardiovascular pathologies. Lysyl oxidase could modulate virtually all stages of the atherosclerotic process, from endothelial dysfunction and plaque progression to calcification and rupture of advanced and complicated plaques, and contributes to vascular stiffness in hypertension. The alteration of LOX/LOXLs expression underlies the development of other vascular pathologies characterized by a destructive remodeling of the ECM, such as aneurysm and artery dissections, and contributes to the adverse myocardial remodeling and dysfunction in hypertension, myocardial infarction, and obesity. This review examines the most recent advances in the study of LOX and LOXLs biology and their pathophysiological role in cardiovascular diseases with special emphasis on their potential as therapeutic targets.

## 1. Introduction

Lysyl oxidase (LOX) is a copper-dependent enzyme which has been classically involved in the oxidative deamination of specific ε-amino groups of lysine and hydroxylysine residues in collagen and elastin, allowing the formation of highly reactive allysine aldehydes and producing hydrogen peroxide as by-product [1,2]. This reaction initiates the covalent cross-linking of collagen and elastin, which is responsible for the tensile strength and elastic properties of connective tissues.

**Figure 1:**
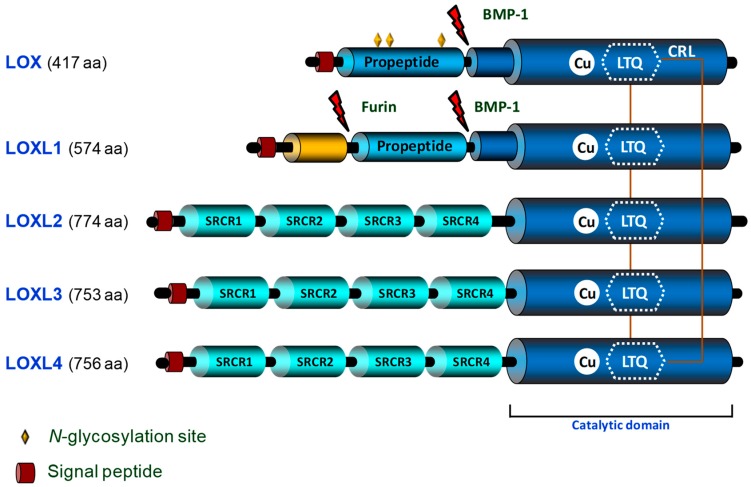
Schematic domain structure and homology of human LOX isoenzymes. The C-terminal regions of LOXs correspond to the highly conserved catalytic domain, which contains the copper binding motif and the lysyl tyrosyl quinone (LTQ) cofactor required for protein conformation and catalytic activity, respectively. Furthermore, the C-terminal domain contains the cytokine receptor-like (CRL) domain. LOX and LOXL1 pro-peptide regions and (Bone morphogenetic protein-1 (BMP-1) cleavage sites, which allow the proteolytic processing of these isoenzymes releasing the active forms, are indicated. The *N*-glycosylation sites in LOX pro-peptide are marked with diamonds. The four scavenger receptors cysteine rich (SRCR) domains contained by LOXLs isoenzymes in the *N*-terminal region are outlined.

**Figure 2:**
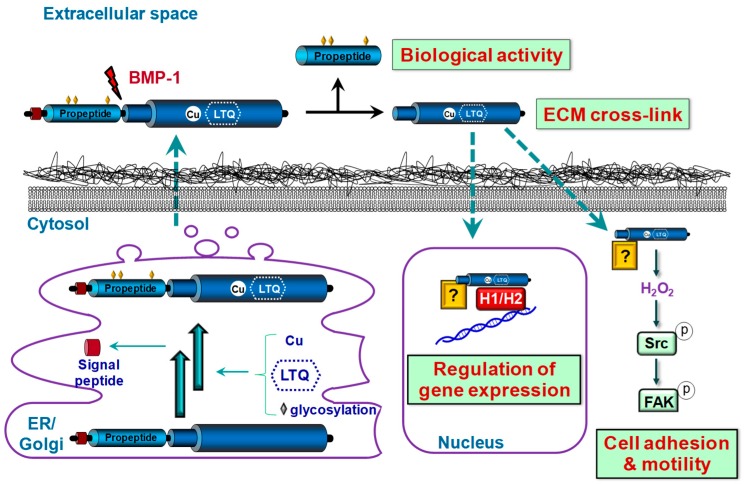
Biological functions and synthesis of LOX. LOX is synthesized as a pre-proenzyme which is post-translationally modified in endoplasmic reticulum (ER) and Golgi. After signal peptide cleavage, copper incorporation, lysyl tyrosyl quinone (LTQ) formation, and glycosylation, the pro-LOX form is released into the extracellular space. Then it is proteolyzed by bone morphogenetic protein (BMP-1) yielding the mature catalytic LOX form, involved in extracellular matrix (ECM) stabilization, and its pro-peptide, which exerts biological functions independently of LOX enzymatic activity. Extracellular LOX could translocate into intracellular compartments by unknown mechanisms. Cytosolic LOX forms participate in the control of cell adhesion and motility in cancer cells, through a mechanism involving the H_2_O_2_-dependent activation of Src-kinase and the subsequent phosphorylation of focal adhesion kinase (FAK). Nuclear LOX regulates gene expression, at least in part, using histones H_1_/H_2_ as substrates.

The LOX family is comprised of five closely related copper-dependent enzymes, LOX, the archetypal member of this family, and four LOX-like isoenzymes (LOXL1, LOXL2, LOXL3, and LOXL4) [3]. These enzymes share a conserved C-terminal region corresponding to the catalytic domain, which consists of the lysine tyrosylquinone (LTQ) cofactor, the copper-binding site, and the cytokine receptor-like domain. The N-terminal regions of these enzymes are structurally unrelated, and their functions are undefined. LOX and LOXL1 contain a basic pro-peptide (PP) region, while LOXL2, LOXL3, and LOXL4 possess four tandem repeats of the scavenger receptor cysteine-rich (SRCR) domains, whose biological function remains unclear (Figure 1). LOX and LOXL1 are synthesized and secreted into the extracellular space as inactive precursors. Then, they are proteolytically processed by bone morphogenetic protein-1 (BMP-1) and other procollagen C-proteinases yielding the catalytically active LOX/LOXL1 forms and their corresponding PPs, in a process in which other extracellular matrix (ECM) components such as fibronectin also participate [4,5] (Figure 2). Whether LOXL2–4 could require a proteolytic processing for their activation is currently unclear.

Beyond ECM maturation, in the last years novel biological functions have been ascribed to LOX and LOXLs (LOX/LOXLs), including the control of epithelial to mesenchymal transition, cell proliferation, migration, adhesion, transformation, and transcriptional regulation. Further, in view of the particular tissue expression patterns of LOX and LOXLs and the phenotypic differences of knockout animal models, it is considered that these isoenzymes would play different physiological roles and that they probably exhibit a dissimilar specificity of substrate. The existence of active intracellular LOX/LOXLs forms, in both the cytoplasm and nucleus, and the biological activity displayed by LOX-PP adds a further degree of complexity and hinders a better knowledge about the role of the LOX family on health and disease [2].

**Figure 3:**
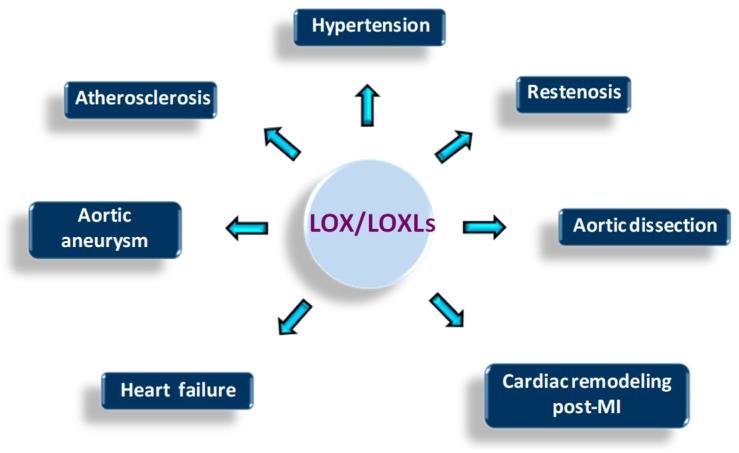
Cardiovascular diseases associated with LOX/LOXLs dysregulation. The disturbance of LOX/LOXLs could underlie the development of several cardiovascular diseases.

Dysregulation of LOX/LOXLs expression/activity has been linked to several human pathologies including cardiovascular diseases (Figure 3). LOX/LOXLs are expressed in the vascular wall and the heart, and the phenotype of genetically modified animal models for these enzymes support their critical contribution to cardiovascular function and development. LOX knockout mice, which die perinatally, show severe vascular abnormalities, characterized by the presence of aortic aneurysms, aortic tortuosity, extensive fragmentation of elastic fibers, morphological and adhesive alterations of endothelial cells, and discontinuities in the smooth muscle cell layer. LOXL1 deficiency is not lethal, but these animals also evidence vascular defects, although not as severe as those detected in LOX^−/−^ mice [6]. Moreover, partial perinatal lethality is observed in LOXL2^−/−^ mice associated with heart defects [7]. While no apparent alterations were detected in heart and aorta from LOXL3 knockout mice [8] and LOXL4^−/−^ are not yet available, recent reports suggest their participation in vascular remodeling [9,10]. This review summarizes the current knowledge about the contribution of the LOX family to cardiovascular diseases, the molecular mechanisms involved in, and discusses their potential interest as pharmacological targets.

## 2. Regulation of Vascular Homeostasis by LOX/LOXLs: Pathophysiological Impact

**Figure 4:**
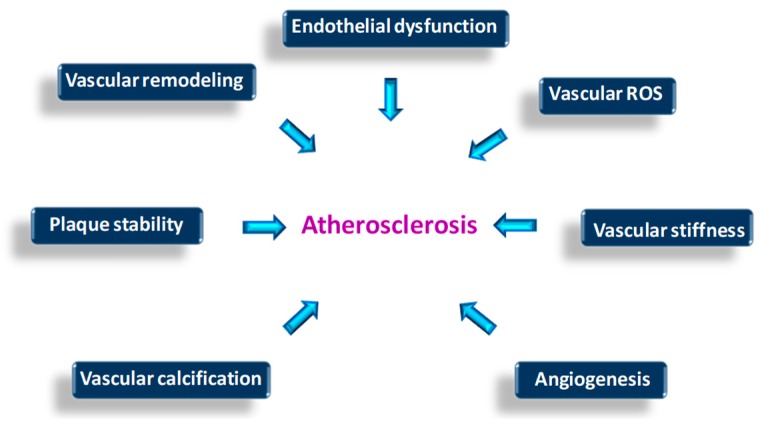
Putative effects of LOX/LOXLs disturbance on the onset and progression of atherosclerosis. The indicated processes could be influenced by LOX/LOXLs dysregulation and affect atherosclerosis progression.

Different mechanisms link LOX virtually to all stages of the atherosclerotic process, from endothelial dysfunction and plaque progression in the early stages, to calcification and rupture of advanced and complicated plaques (Figure 4). LOX dysregulation seems also to be involved in other vascular pathologies related with atherosclerosis, but mainly characterized by an intense destructuration of ECM such as aneurysms and artery dissections.

### 2.1. Contribution to Arterial Aneurysms and Aortic Dissections

An aneurysm is a vascular abnormality characterized by the weakness and local dilation of the arterial wall, defined as a 50% increase in the normal diameter of the vessel. Aortic aneurysms are the most common form of this disease, a severe condition with high morbidity and mortality which can lead to vascular rupture or dissection [11].

The potential involvement of LOX/LOXs in aortic aneurysm was already suggested in the 1990s, when different experimental approaches in animal models supported the importance of LOX in the maintenance of the mechanical stability of the aorta [12,13,14]. In fact, in rats, the irreversible inhibition of LOX/LOXL activity with β-aminopropionitrile (BAPN) promoted aortic dilation [12]. Further, the administration of BAPN to angiotensin II (Ang II)-infused ApoE knockout mouse, a classical model of aortic abdominal aneurysm (AAA), enhanced AAA incidence [15]. Interestingly, in two other experimental models of AAA in rodents, the elastase- and the CaCl_2_-induced AAA models, aneurysm development was accompanied by a reduction of LOX expression and activity [13,14]. It should be highlighted that local overexpression of LOX by aortic adenoviral delivery limited the development of established CaCl2-induced AAA, at least in part by inhibiting vascular smooth muscle cells (VSMC)-mediated MCP-1 secretion and JNK activity [14,16]. Similarly, in mice, genetic inactivation of LOX, but not that of LOXLs, leads to aortic aneurysms and spontaneous dissections, supporting that the inhibition of this specific isoenzyme could underlie the development of this disease [17,18].

Although experimental data support that LOX inhibition underlies AAA, in humans there is no solid evidence showing a direct causal relationship between LOX/LOXLs and aortic aneurysms, probably because human tissue specimens often come from late stages of aneurysmal disease. Considering that AAA is a focal manifestation of a generalized vascular disturbance [19], LOX has been found upregulated in a recent proteome analysis of internal mammary arteries from patients with AAA [20]. The authors consider the increase in LOX expression a compensatory response triggered by paracrine or endocrine mechanisms to stabilize the aortic wall. Whether LOX activity/expression fails at early stages of human AAA is difficult to assess because of the lack of vascular samples of these stages of the disease.

Regarding thoracic aortic aneuryms (TAA), in 2016 two independent studies simultaneously identified both a loss of function mutation and rare genetic variants in LOX predisposing to TAA [21,22]. Further, LOX protein levels were found downregulated in aortic dissections from ascending aorta compared with healthy specimens [23], in agreement with a previous single case report study [24].

While defective LOX levels have been related to nonsyndromic aneurysms of ascending aorta, enhanced LOX and LOXL1 expression and higher LOX activity have been reported in the aorta from animal models and patients with Marfan syndrome, a systemic connective tissue disorder characterized by severe cardiovascular manifestations, including TAA, acute aortic dissections, and aortic ruptures [25]. The authors argue that the enhanced LOX-dependent collagen deposition and cross-linking in this disease should be regarded as a compensatory response aiming to preserve aortic integrity.

A reduction of vascular LOX expression has also been described in patients with cerebral aneurysm and in experimental models of this disease, an effect associated with the high vascular levels of interleukin-1β and the activation of NFĸB signaling characteristics of this disorder [26]. A single nucleotide polymorphism of LOXL2 has been associated with cerebral aneurysms [27], suggesting that the inhibition of LOX/LOXLs could be a key event in the pathophysiology of this disease.

Therefore, aneurysmal diseases are associated with suppression of vascular LOX activity, while experimental interventions or pathophysiological compensatory responses increasing LOX activity seem to preserve artery integrity. Altogether, these data indicate that therapeutic approaches aiming to normalize LOX activity could preserve the mechanical properties of the aortic wall and be a promising strategy to limit the progression of different forms of vascular aneurysms.

### 2.2. LOX in Endothelial Dysfunction

LOX is highly expressed in the endothelium of healthy arteries [28], and multiple evidence associates LOX downregulation with endothelial dysfunction. Indeed, endothelial LOX expression is strongly inhibited by different conditions, which trigger endothelial dysfunction including high levels of low-density lipoproteins (LDL) [29], hyperhomocysteinemia [30], and pro-inflammatory cytokines [31]. LDL increased the exchange of macromolecules across an endothelial monolayer, similarly to the irreversible LOX inhibitor BAPN, suggesting that LOX downregulation by LDL could impair endothelial barrier function [29]. The modulation of LOX by atherogenic concentrations of LDL operates both in vitro and in vivo and has been observed in endothelial cells as well as in VSMC [28,29]. Although the mechanism has not been characterized in detail, it seems to require the internalization of LDL by the LDL receptor (LDLR) and the subsequent lysosome-mediated processing [28]. Hyperhomocystinemia, an atherosclerotic risk factor that induces endothelial dysfunction and alters the elastic properties of the vascular wall, also inhibits LOX activity and negatively regulates endothelial LOX expression at the transcriptional level [30]. The detrimental effects of homocysteine on LOX activity have also been observed in small vessels in proliferative diabetic retinopathy [32]. The inhibition of LOX by homocysteine depends on the thiol-group and the generation of reactive oxygen species (ROS) [30]. Although the mechanism has not been further investigated in endothelial cells, in other cell types it involves IL-6, Fli1, and epigenetic CpG methylation [33]. Likewise, pro-inflammatory cytokines impair endothelial function and reduce LOX expression and activity both in vitro and in vivo [31]. TNFα downregulation of LOX mRNA levels and enzymatic activity in endothelial cells depends on TNFR2 and seems to be the consequence of a decrease in LOX transcriptional activity. Interestingly, statins, lipid-lowering drugs widely used in the management of vascular diseases that improve endothelial function, normalize vascular LOX down-regulation induced by TNFα in vitro and by hypercholesterolaemia in vivo [34]. Although inducers of endothelial dysfunction commonly downregulate LOX, in human endothelial cells LOX is upregulated by advanced glycation end products (AGEs) via RAGE/MAPK signaling and NF-κΒ and AP-1, thereby impairing endothelial homeostasis [35]. Taken together these results indicate that LOX dysregulation underlies endothelial dysfunction elicited by cardiovascular risk factors.

### 2.3. LOX in Atherosclerosis Progression and Plaque Instability

The knowledge on the role of LOX in the onset, progression, and instability of atherosclerotic plaque has been hampered by the lack of studies on this subject in humans, but different approaches in animal models support the contribution of LOX in atherosclerosis.

In an early study conducted by Chvapil et al. [36] in very young chickens (1–2 days old) subjected to a short feeding period with a cholesterol-rich diet, vascular LOX activity was not modified. However, in the porcine model fed a high-fat diet (HFD) we observed that hypercholesterolemia downregulated aortic LOX expression in the earliest stages of the atherosclerotic process (fatty streaks in the aorta after 100 days of diet intervention) [29]. By contrast, in advanced atherosclerosis induced in rabbits fed an atherogenic diet, LOX activity significantly increased, particularly in the aortic arch [37]. In aortic atherosclerosis-prone regions of the ApoE-deficient (ApoE-KO) mice, the expression of LOX as well as several genes encoding for ECM components, such as collagen I and fibronectin, were increased [38]. When these animals were switched to a HFD, despite the extraordinary high plasma cholesterol levels reached, inhibition of LOX by the administration of BAPN reduced atherosclerotic lesion formation (≈50%). Second harmonic generation 2-photon microscopy revealed reduction in structured collagen in the neointima of lesions from BAPN-treated animals, with no changes in total collagen I [38]. Further, macrophage abundance in lesions was reduced by BAPN, an effect that was related to the low adhesiveness of monocytes/macrophages to the ECM from these animals observed in vitro. Finally, in a recent study, intense LOX immunostaining was located surrounding calcified areas in advanced atherosclerotic lesions from humans [39] (see Section 2.5).

In complicated atherosclerotic plaques, LOX activity could impact on plaque stability. The stability of the atherosclerotic plaque is preserved by the fibrous cap that overlies the lipid core. Acute clinical complications, such as myocardial infarction, are usually caused by a thrombus formed on a vulnerable plaque with a weakened fibrous cap that breaks. Fibrillar collagens synthesized by VSMC are the main components of the fibrous cap, whose biomechanical properties and strength depends on the cross-linking of collagen fibers [40]. In this regard, low LOX activity could lead to defective collagen cross-linking, which would weaken the fibrous cap and favor the presence of soluble forms of collagen highly susceptible to metalloproteinase degradation. In a recent study in atherosclerotic plaques from human carotid endarterectomy specimens, LOX was strongly expressed in areas with ongoing fibrogenesis, in the deeper layers of the fibrous cap and around the necrotic core enriched in CD163 + macrophages (M2 macrophages with profibrotic and anti-inflammatory functions) [41]. In this study, higher levels of LOX were detected in more stable plaques and, interestingly, high levels of LOX mRNA in carotid plaques were associated with a lower incidence of myocardial infarction during the follow-up. Mediators of inflammation produced by activated macrophages, T cells, and other inflammatory cells present in unstable plaques may control LOX expression and hence plaque stability. Interferon γ (IFNγ), a cytokine involved in matrix remodeling and induced in ruptured atherosclerotic plaques, reduces collagen I synthesis [42] and downregulates LOX in VSMC by both transcriptional and post-transcriptional mechanisms [43]. Atherosclerotic lesions of mice with increased T-cell activation shows reduced LOX expression and collagen cross-linking, probably due to the inhibition of LOX by cytokines produced by activated T cells [44]. Conversely, in atherosclerosis-prone mice, osteoprotegerin (OPG) promoted VSMC accumulation, LOX upregulation, LOX-dependent collagen fiber maturation, and the formation of stable fibrous caps [45]. Therefore, LOX-dependent collagen cross-linking could play a relevant role in limiting clinical complications by stabilizing atherosclerotic lesions and preventing fibrous cap rupture. It should be taken in mind, however, that the modulation of LOX and LOXLs such as LOXL2 by hypoxia [10,46] and their role in neovascularization, could also indirectly affect plaque stability. Further, recent research has evidenced that mice overexpressing LOX in platelets have more severe thrombosis supporting that LOX enhances platelet activation and thrombosis [47]. Specific studies to better establish the role of LOX/LOXLs in plaque instability are still needed.

### 2.4. LOX in VSMC, Vascular Remodeling, and Restenosis

VSMC plays a key role in vascular remodeling, a process which entails cell migration and proliferation, as well as the production and reorganization of the ECM. LOX has been associated with VSMC migration and proliferation and, as the isoform responsible for the 80% of the LOX activity in aortic VSMC [48], is essential in ECM maturation.

Different growth factors and cytokines that activate VSMC and ECM synthesis increase LOX expression. Transforming growth factor β (TGFβ) increases LOX expression and activity in VSMC [49] and concomitantly enhances the synthesis of ECM components. Platelet derived growth factor (PDGF), a key mitogen that promotes VSMC migration and proliferation and neointimal growth, also increases LOX expression [50]. Both PDGF-BB and TGFβ are involved in the cross-talk between endothelial and VSMC induced by low shear stress leading to LOX upregulation and vascular remodeling [51]. Further, LOX participates in vascular remodeling in HFD-fed rats and mediates the profibrotic effects triggered by leptin-induced TGFβ in VSMC [52]. On the other hand, LOX binding to this cytokine could suppress TGFβ signaling [53]. The vascular expression of LOX also seems to be highly dependent on granulocyte macrophage colony-stimulating factor (GM-CSF). GM-CSF increases the expression of both LOX and BMP-1 in VSMC, and GM-CSF-deficient mice exhibit low levels of LOX, BMP-1, and tropoelastin, and impaired cross-linkage of elastic fibers [54].

**Figure 5:**
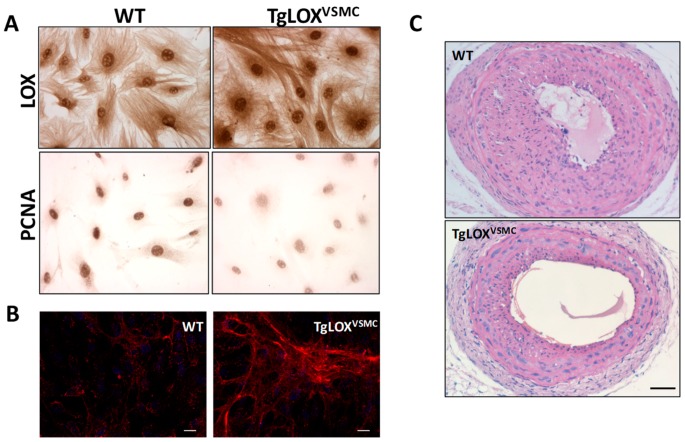
LOX transgenesis limits VSMC proliferation and vascular remodeling. (**A**) Immunocytochemical analysis of LOX and PCNA, markers of cell proliferation, in aortic VSMC from TgLOX^VSMC^ and WT mice. (**B**) Collagen type-I deposition (in red) by VSMC from TgLOX^VSMC^ and WT mice visualized by confocal immunofluorescence in nonpermeabilized cells, as described [39]. Cells were incubated with a COL1A1 specific antibody (NB600-408; Novus Biologicals, UK) and nuclei were stained with DAPI. As observed, LOX transgenesis induces the deposition of a thicker and more organized collagen network (Bar: 25 µm). (**C**) TgLOX^VSMC^ and WT mice were subjected to left common carotid artery ligation. Injured arteries were harvested 21 days after surgery, fixed in 4% paraformaldehyde, and embedded in paraffin. Cross-sections at 1.5 mm from the ligation site were stained with hematoxylin and eosin, as described [62,63]. This staining evidenced that LOX overexpression reduces neointimal growth (Bar: 100 µm).

In the past years, novel findings suggest that LOX/LOXLs functions extend beyond their role in the stabilization of the ECM. Indeed, they markedly influence cell chemotactic responses, proliferation, adhesion and migration [55], all of them cellular processes implicated in vascular remodeling. It has been reported that LOX activity is essential to generate optimal chemotactic sensitivity of VSMC to chemo-attractants by oxidizing specific cell surface proteins, such as PDGF receptor-β (PDGFRβ) [56,57]. Concerning VSMC proliferation, mitogenic stimulation upregulates LOX in rat adult VSMC [49], and a computational method identified LOX as a VSMC gene responsive to proliferative stimulus [58]. In agreement, the expression of LOX and LOXLs increased in pathologies characterized by intense VSMC migration and proliferation. LOX mRNA and protein expression time-dependently increased after balloon injury of the rat carotid artery, preceding maximal collagen accumulation and neointimal thickening [59]. In rabbits undergoing angioplasty, LOX inhibition reduced restenotic rates and constrictive remodeling [60]. Further, all five LOX/LOLXs are dysregulated in clinical and experimental pulmonary hypertension [10], a pathology characterized by increased muscularization due to VSMC migration and proliferation, and perturbed matrix structures in the vessel wall of small pulmonary arteries. However, other studies concluded that LOX restrains VSMC proliferation. Indeed, an inverse correlation between LOX expression and neonatal VSMC proliferation has been reported [50]. LOX-PP also inhibited the proliferation of VSMC in culture, and was detected at high levels in vascular lesions of injured arteries, suggesting that it could be part of a feedback mechanism to limit vascular remodeling [61]. Recently, in a transgenic mouse which overexpresses LOX in VSMC (TgLOX^VSMC^) we have shown that cell proliferation was significantly reduced in TgLOX^VSMC^ VSMC [62]. Transgenic VSMC also exhibited low levels of Myh10 (marker of SMC phenotypic switching), PCNA (marker of cell proliferation), and MCP-1, and a reduced activation of Akt and ERK1/2 in response to mitogenic stimuli (Figure 5A). Accordingly, neointimal thickening induced by carotid artery ligation was attenuated in TgLOX^VSMC^ mice (Figure 5C). In vivo and in vitro studies ruled out any contribution of LOX-PP to the antiproliferative activity of LOX on VSMC and evidenced the participation of extracellular and enzymatically active LOX forms [63].

Finally, enzymatically active forms of LOX have been detected in the nucleus of VSMC [64,65]. The biological activity of nuclear LOX remains unraveled, but by affecting chromatin organization they could modulate gene expression, as has been shown for ECM components such as elastin and collagen [66,67,68], and VSMC proliferation [69].

### 2.5. LOX in Vascular Calcification

In the last decades, several epidemiological studies have demonstrated that vascular calcification is an independent predictor of cardiovascular events and all-cause mortality in the general population [70,71,72]. Vascular calcification is a complex and tightly organized biological process characterized by the deposition of calcium phosphate, the phenotypic transdifferentiation of vascular cells into osteo/chondroblast-like cells and an active remodeling of the ECM, which seems to play an active role in this process. Our recent investigations points towards LOX as a fundamental player in vascular mineralization. Using gain- and loss-of-function approaches we uncovered that LOX contributes to the hyperphosphatemia (HPM)-induced calcification of VSMC and to VSMC-to-osteoblast commitment [39]. In fact, HPM-induced VSMC calcification was accompanied by an up-regulation of LOX expression in both human and murine VSMC. VSMCs isolated from TgLOX^VSMC^ mice exhibited an exacerbated HPM-induced calcium deposition and a higher increase in the expression of osteogenic factors than wild-type cells. Similarly, in ex vivo studies on aortic rings from these animals exposed to osteogenic media, we evidenced that LOX transgenesis was accompanied by enhanced deposition of calcium. In line with the increased expression of LOX found in human VSMC cultured under osteogenic conditions, we observed that HPM-induced mineralization was associated to a stronger deposition of insoluble collagen [39], highlighting the importance of ECM quality on this process. Moreover, pharmacological inhibition of LOX using BAPN (or LOX knockdown, by RNA interference) limited calcium deposition and the upregulation of osteogenic factors induced by HPM in human VSMC. In contrast with our data, the use of β-glycerophosphate as an inducer of VSMC calcification in bovine VSMC promotes a downregulation of LOX, discrepancies which are probably due to species-specific responses and disparities in the transduction pathways activated by each procalcifying stimulus [73]. Of note, in human atherosclerotic lesions we found a positive relationship between LOX expression and calcium burden, and similarly enhanced LOX expression and collagen cross-linking have been reported in human calcific aortic valve disease, supporting the pathophysiological relevance of our results [39,74]. Considering that vascular calcification is a determinant factor of vascular stiffness, our data evidence the critical involvement of LOX in both processes (see below).

### 2.6. LOXs, Vascular Stiffness, and Oxidative Stress: Impact on Hypertension

Vascular stiffening is a hallmark of vascular aging and pathological processes such as diabetes atherosclerosis and hypertension. It has been established that vascular stiffness, measured as aortic pulse wave velocity (aPWV), is an independent predictive factor of cardiovascular events both in the general population and in subjects with other risk factors [75,76]. Therefore, the control of vascular stiffness could be a promising strategy to ameliorate cardiovascular mortality, especially in hypertension, because arterial stiffening itself has been proposed to be a causal factor in essential hypertension [77].

**Figure 6:**
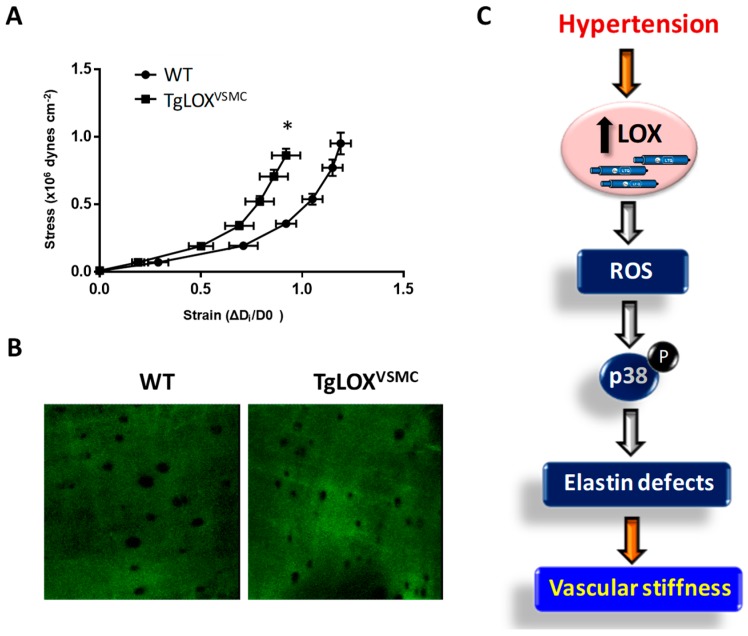
Contribution of LOX to oxidative stress and vascular stiffness in hypertension. (**A**) The structural and mechanical properties of first-order mesenteric arteries were studied with a pressure myograph (Danish Myo Tech, Aarhus, Denmark) as previously described [78,79]. Stress–strain curves were obtained in arteries from WT and TgLOX^VSMC^ mice. Vascular stiffness was determined from the stress–strain relationship which is nonlinear; therefore, we obtained the incremental elastic modulus (Einc) by determining the slope of the stress–strain curve of individual animals. Einc values were (mean ± SEM) WT: 4.11 ± 0.12 (*n* = 6); LOX: 5.17 ± 0.27 (*n* = 8). *p < 0.05 vs. WT by unpaired student t-test. For simplification, statistical analysis is shown in the stress–strain curves. (**B**) The elastin organization within the internal elastic lamina was studied in segments of mesenteric arteries from TgLOX^VSMC^ and WT mice using fluorescence confocal microscopy based on the autofluorescent properties of elastin (ex: 488 nm; em: 500–560 nm), as previously described [78,79]. Serial optical sections from the adventitia to the lumen (z step = 0.5 µm) were captured with a X63 oil objective, by using the 488 nm line of the confocal microscope. (image size 53 × 53 µm). (**C**) Working model showing that LOX induced-oxidative stress alters elastin structure and promotes vascular stiffness in hypertension.

Vascular stiffness is influenced, among other factors, by the structural elements of the arterial wall, being collagen and elastin particularly determinants. Given the central role of LOX in collagen and elastin cross-linking, we have recently assessed the contribution of LOX to vascular stiffness and hypertension [78]. Mesenteric resistance arteries from TgLOX^VSMC^ were stiffer than those from control animals (Figure 6A). This effect was associated with a decrease in the number and size of fenestrae in the internal elastic lamina (Figure 6B), alterations which have been previously associated to the increased vascular stiffness in animal models of hypertension or during development [79]. The increased vascular stiffness and the disturbance on elastin structure found in TgLOX^VSMC^ mice were ameliorated by BAPN, indicating that these alterations are due to the enhanced vascular LOX activity exhibited by these mice. Additionally, these animals showed an increased production of H_2_O_2_, the by-product of LOX activity, along with a rise in vascular O_2_^.−^ levels, NADPH oxidase activity, NOX-1 expression, and mitochondrial dysfunction [78]. These results evidenced that LOX is a novel source of vascular oxidative stress. It is worth mentioning that the LOX-induced production of ROS is responsible for the enhanced vascular stiffness and the elastic alterations exhibited by LOX transgenic mice, since these disturbances were attenuated by an H_2_O_2_ scavenger and a mitochondria-targeted antioxidant [78].

It should be noted that although TgLOX^VSMC^ mice are normotensive, their vascular alterations resemble those from hypertensive models. In fact, our investigations also revealed that LOX is upregulated in the vascular wall of two animal models of hypertension, the spontaneously hypertensive rat (SHR) and Ang II-infused mice, and that the increase in vascular LOX levels results from the hemodynamic effect induced by hypertension. More interestingly, the upregulation of LOX in hypertension is partially responsible for the enhanced vascular stiffness and the higher production of ROS from different sources found in these hypertensive models, as revealed in experiments in which LOX activity was inhibited by BAPN. Likewise, BAPN also abolished the Ang II-induced activation of p38MAPK, responsible for vascular stiffness in hypertension. In accordance, TgLOX^VSMC^ mice exhibit an activation of p38MAPK in the vessel wall, which relies on the higher vascular oxidative stress of these animals, while the inhibition of this kinase limited stiffness and the alterations in elastin structure [78]. Therefore, using complementary gain-of-function and LOX inhibition approaches, we demonstrated that LOX up-regulation is associated with enhanced vascular oxidative stress, which promotes p38MAPK activation, elastin structural alterations, and vascular stiffness in resistance and conductance arteries in hypertension (Figure 6C).

While our study was focused on elastin, both collagen and elastin cross-linking could contribute to vascular stiffness in hypertension as has been suggested in Ang II-infused animals [80]. In fact, in Ang II-infused mice, BAPN reduced vascular collagen content and cross-linking and vascular stiffness. Then, we cannot discard that the higher vascular stiffness exhibited by TgLOX^VSMC^ mice could be associated with the enhanced deposition of mature collagen that we described in this animal model (Figure 5B) [62]. In any case, our study provides novel information about the key role of LOX in elastin deposition and vascular stiffness.

Although the participation of LOX in vascular stiffness is well demonstrated, its contribution to high blood pressure is still unclear. TgLOX^VSMC^ mice (at 3 months of age) show normal blood pressure, and although BAPN partially prevented hypertension development in Ang II-infused mice, it was not able to decrease high blood pressure in adult SHR with well-established hypertension [78]. Moreover, newborn LOX^-/-^ mice show normal blood pressure [81]. It is important to note that arterial stiffening is not the unique determinant of hypertension, but many other mechanisms might operate, and therefore, the contribution of LOX should be specifically addressed. Nevertheless, our results indicate that limiting ECM cross-linkage could ameliorate vessel stiffness and improve the management of patients with cardiovascular disorders.

Regarding other isoenzymes of this family, LOXL2 was identified as a candidate gene in a genome-wide association study for blood pressure and arterial stiffness [82]. Indeed, this enzyme has been recently involved in the regulation of age-associated vascular stiffness, since the increase in PWV induced by aging was ameliorated in LOXL2^+/−^ mice [83]. The authors demonstrated that LOXL2 enhances VSMC stiffness and contractility and induces matrix deposition. These results are in agreement with previous reports supporting that the intrinsic stiffening of VSMC also underlies the enhanced aortic stiffness with aging [84]. Whether LOX and other LOXL isoenzymes are involved in the control of intrinsic VSMC stiffening is an issue that deserves further research.

### 2.7. LOX and LOXLs in Neovascularization

Both LOX and LOXL2 have been positively involved in neovascularization, although most of the approaches were focused on tumor angiogenesis. In endothelial cells, the expression of these enzymes is induced by hypoxia, which is a potent angiogenic stimulus [46,85]. In fact, in models of pathological and developmental neovascularization, LOXL2 was detected in angiogenic endothelial cells and tip cells [85,86] and its knockdown in zebrafish embryos significantly suppressed intersegmental vessel circulation. Gain- and loss-of-function studies in HUVEC and microvascular endothelial cells evidenced that LOXL2 contributes to endothelial tube formation through the modulation of endothelial cell migration and proliferation [85,87], and more importantly through collagen IV network assembly, which seems to be critical for capillary formation [85]. It should be noted that in this study on cell migration, tubulogenesis and collagen IV assembly were modestly affected by BAPN, and that a catalytically inactive LOXL2 mutant triggered a similar increase in endothelial cell migration and angiogenic sprouting than wild-type LOXL2. Thus, this suggests that the modulation of these processes by LOXL2 only partially depends on its catalytic activity [85,87]. Indeed, it has been recently suggested that LOXL2 might promote angiogenesis through the regulation of endothelial-to-mesenchymal transition (EndMT) and the activation of PKB/AKT and FAK signaling pathways [87]. However, the contribution of LOXL2 enzymatic activity could not be ruled out because a LOXL2 inhibitory antibody (AB0023) ameliorated basic fibroblast growth factor (bFGF)-induced cell migration and network assembly in HUVEC [88]. The expression of LOXL2 was also specifically induced in tumor endothelial cells which LOXL2 blockade with AB0023 limited tumor vascularization [88,89]. Likewise, small LOXL2 inhibitors decrease tumor angiogenesis and restrict tumor growth [90].

While LOXL2 has been involved in the angiogenic response of both normal and tumoral endothelial cells, evidence regarding the contribution of LOX to neovascularization is weak and the studies were mainly focused on tumor angiogenesis. Tumor-derived LOX drives angiogenesis through PDGFRβ stimulation and AKT activation leading to an increased production of VEGF [91]. However, it should be noted, that this proangiogenic response was exerted by the LOX-induced production of VEGF by tumor cells and that whether this mechanism could operate in vascular endothelial cells in an autocrine manner was not evaluated. A subsequent study evidenced that LOX expression and activity are sharply higher in tumor endothelial cells than in normal cells and that LOX knockdown limits tumor endothelial cell motility and tubulogenesis through the inhibition of FAK signaling [92]. Further, overexpression of both LOX and LOXL2 increased tumor perfused vessel density in different syngeneic models [93]. In contrast, in a mouse model of breast cancer, while the number of tumor-associated endothelial cells was reduced by the LOXL2 targeted antibody AB0023, microvessel density was not significantly modulated by M64, a LOX-specific antibody raised against LOX catalytic domain, supporting a major role of LOXL2 on tumor neovascularization [89].

A recent study reported that LOX-PP inhibits major angiogenic signaling pathways, attenuating HUVEC proliferation, migration and adhesion, and reducing tubulogenesis in the chicken chorioallantoic membrane assay [94]. Although high concentrations of LOX-PP were used in some of these assays, in view of these data, further studies should be necessary to clarify the role of each specific LOX form in physiological and pathological angiogenesis.

In the last years, exosomes raised as key signaling players in the cross-talk between different cell types, as carriers of mRNAs, miRNAs, and proteins. It has been described that LOXL4 could be delivered by hepatocellular carcinoma exosomes and transferred to endothelial cells promoting angiogenesis [95]. Similarly, glioma cell-derived exosomes are internalized by endothelial cells. Exosomes from glioma cells exposed to hypoxia were enriched in LOX and in other relevant proteins in angiogenesis inducing microvascular sprouting. Whether the cargo of LOX in hypoxic exosomes could actively contribute to their pro-angiogenic activity should be, however, clarified [96,97]. Based on these studies and others reporting that LOXL2 levels were increased in endothelial cell-derived exosomes from patients with atherosclerotic cerebrovascular disease, it is tempting to speculate that exosome-derived LOX/LOXLs could have a relevant pathophysiological role in cardiovascular and tumor diseases.

## 3. LOX and Heart Disease

Myocardial interstitial fibrosis is a hallmark of virtually all forms of heart disease, having detrimental consequences on left ventricular (LV) function and actively contributing to the progression of heart failure (HF), a leading cause of death in industrialized countries. Fibrosis, which is a critical determinant of diastolic dysfunction and cardiac stiffness, is characterized by an unbalance between synthesis and degradation of collagen, the major constituent of cardiac ECM. Enhanced cardiac LOX/LOXLs expression/activity has been associated with the adverse myocardial remodeling and dysfunction in animal models of hypertension, cardiac infarct, and obesity [52,98,99,100]. Interestingly, in the last years different studies in both hypertensive animal models and humans have highlighted the importance of collagen quality beyond collagen amount negatively influencing heart function [99,101,102,103]. In hypertensive patients with stage C chronic HF, elevated filling pressures are associated with the degree of collagen cross-linking (but not with total collagen) which, in turn, correlates with cardiac LOX expression and more importantly, with an impairment of systolic and diastolic function [102]. In agreement, HF patients with normal ejection fraction showed enhanced myocardial collagen content, but also increased LOX expression and collagen cross-linking, both associated with diastolic dysfunction [103]. The relationship between LOX and diastolic function was also evidenced in a recent study using a new LOX transgenic mouse model. This work shows that myocardial LOX overexpression in mice is associated with an age-dependent disturbance of diastolic function and an accelerated cardiac remodeling [104]. Interestingly, LOX transgenesis also exacerbates Ang II-induced cardiac hypertrophy and systolic dysfunction, enhancing inflammation and ROS production and altering Ang II-dependent signaling. Further, this study uncovers the ability of LOX to promote fibroblast-to-myofibroblast transition, a critical process involved in cardiac fibrosis and remodeling. Similarly, the expression of LOXL2 is increased in patients with ischemic or idiopathic dilated cardiomyopathy in which cardiac LOXL2 correlates with collagen cross-linking and with the impairment of diastolic function [105]. Consistent with data described above for LOX, LOXL2 also contributes to cardiac fibroblast reprogramming [105], highlighting the importance of ECM stiffness and composition driving myofibroblast activation [106]. Therefore, these data evidence that the upregulation of LOX/LOXLs in cardiac diseases not only passively contribute to cardiac fibrosis, but actually implies that these enzymes are active players in cardiac dysfunction and disease progression.

Mechanistically, most of the growth factors and cytokines involved in cardiac fibrosis could contribute to the upregulation of LOX and LOXLs in the pathological myocardium. TGFβ, a critical cytokine involved in cardiac fibrosis and remodeling, is an upstream regulator of LOX in cardiac fibroblasts, inducing LOX expression through PI3K/Akt, Smad3, and MAPKs signaling pathways [107]. The enhanced Smad2/3 signaling triggered by TGFβ also increases c-jun expression which is the ultimate responsible for the TGFβ-mediated activation of LOX transcription [108]. Concomitantly, this response is associated to an enhanced expression of collagens I and III, and BMP-1, resulting in a greater fibrotic response [107]. Similarly, inflammatory factors have been implicated in adverse cardiac remodeling and fibrosis, including TNFα and osteopontin (OPN). Transgenic mice that overexpress TNF-α in the myocardium develop cardiac hypertrophy, fibrosis, dilated diopathy, and premature death [109]. The profibrotic response triggered by TNFα in the myocardium involves the upregulation of LOX due to a TNFα-dependent induction of TGFβ signaling [110]. In turn, OPN, a multifunctional cytokine strongly expressed in the fibrotic myocardium, promotes fibroblast-to-myofibroblast transdifferentiation and upregulates LOX in human fibroblasts. The authors suggest that OPN contributes myocardial stiffness and dysfunction in patients with hypertensive heart disease and HF, at least in part, through the upregulation of LOX and the consequent increase in collagen crosslinking [111]. Likewise, the activation of the TH1 lymphocytic function in mice enhances myocardial LOX expression (LOX and LOXL3) and activity and collagen crosslinking, thereby altering cardiac diastolic function [112]. Although significant efforts have been made to decipher the molecular mechanisms underlying the enhanced ECM crosslinking and LOX/LOXLs expression in the diseased heart, further studies should be addressed to clarify this issue.

Considering the negative impact of an excessive LOX/LOXL-mediated crosslinking of collagen on cardiac function, pharmacological strategies targeting LOX/LOXLs raise as promising therapeutic tools in patients with HF, as discussed below.

## 4. LOXs as Pharmacological Targets for Cardiovascular Diseases

As outlined in this review, the impact of LOX/LOXLs dysregulation on the cardiovascular system and its contribution to different cardiovascular disorders support the interest of these enzymes as novel pharmacological targets for these diseases. The development of LOX/LOXLs-targeted therapeutic tools, however, has been hampered by the unavailability of their crystallographic structure and the fact that both increases and decreases of LOX/LOXLs underlie the development of different cardiovascular diseases that often coexist. Therefore, the therapeutic blockade or, when appropriate, the upregulation of LOX/LOXLs must be thoroughly evaluated to ensure their safety.

The importance of collagen quality in heart performance and the impact of LOX/LOXLs on cardiac fibroblast function highlight the interest of LOX/LOXLs-inhibitory therapies to slow HF progression and limit the detrimental impact of cardiac remodeling in response to myocardial infarction [98,101,102,103]. Further, an excessive LOXs-dependent cross-linking could interfere with tissue drug distribution, as demonstrated for chemoresistant tumors [93] and, therefore, limiting LOX-induced cardiac stiffness could improve the therapeutic response to established therapies. However, chronic and irreversible LOXs inhibition, such as that achieved by BAPN, might disturb the appropriated ECM scaffold and compromise ventricular integrity. Similarly, although LOX inhibition could decrease vessel stiffness and this might have beneficial effects to protect from cardiovascular events in hypertension, chronic LOX/LOXLs blockade could promote plaque instability, while the risk of vascular aneurysm and dissection should not be ignored. It is clear that irreversible inhibitors of LOX/LOXLs, such as BAPN, have enormous limitations as therapeutic tools for chronic use. In fact, it has been reported that chronic administration of BAPN induces neurotoxicity and causes lathyrism, a disorder characterized by connective tissue alterations which notably affects bone mechanical strength and blood vessels [113,114,115,116]. To avoid these drawbacks, monoclonal antibodies against LOX and LOXL2 have been developed and proven effective in reducing cardiac fibrosis and dysfunction in experimental models of pressure overload and myocardial infarct [98,105]. Simtuzumab, a humanized IgG4 monoclonal antibody against LOXL2, has been tested for its antifibrotic capacity in patients with liver and lung fibrosis and pancreas cancer [117]. However, these clinical trials fail to evidence the efficacy of simtuzumab as an antifibrotic agent despite promising results in preclinical studies in animal models [117]. Whether simtuzumab could provide positive results limiting cardiac fibrosis is currently unknown. Likewise, small molecules that directly inhibit LOXL2 catalytic activity are being developed and tested into healthy volunteer Phase 1 trials [118].

Beyond the development of specific LOX/LOXLs inhibitors, other strategies to reduce LOX activity might involve the use of chelating agents, such as bathocuproine or polyamine chelators, which limit the availability of copper required for the biogenesis of LTQ. Alternatively, BMP-1 inhibition, which would limit the extracellular processing of LOX and LOXL1, could be an additional approach to inhibit LOX.

Another issue to be considered is the potential impact on LOX activity of drugs in use for the management of cardiovascular diseases. Current HF therapies such as torasemide reduced cardiac LOX expression, collagen cross-linking, fibrosis, and LV stiffness in patients with hypertensive HF [119]. Similarly, EXP3179, a metabolite of losartan, reduced cardiac LOX expression and collagen cross-linking in hypertensive rats [120], while, as described above, statins normalize endothelial LOX expression in hyperlipemic animals [34].

## 5. Conclusions and Future Challenges

The experimental findings summarized in this review evidence the key contribution of LOX and LOXLs to multiple aspects of cardiovascular homeostasis, which could be relevant for the development of high-incidence human diseases such as atherosclerosis, HF, arterial aneurysms and aortic dissection. Although significant advancements have been made, overall, the failure of clinical trials in analyzing the antifibrotic properties of simtuzumab indicates the need of a deeper understanding of LOX/LOXLs biology. It is mandatory to identify which are their specific intracellular and extracellular functions and substrates, to develop novel methods allowing to assess the degree of LOX inhibition in the targeted tissues and to identify those patients who will benefit by LOX/LOXLs-targeted therapy. Because regulation of LOX activity could be a double-edged sword, safety concerns should be seriously approached.
